# Enhanced Practical Byzantine Fault Tolerance via Dynamic Hierarchy Management and Location-Based Clustering

**DOI:** 10.3390/s24010060

**Published:** 2023-12-21

**Authors:** Gwangyong Kim, Jinsung Cho, Min Choi, Bongjae Kim

**Affiliations:** 1 Department of Computer Engineering, Chungbuk National University, Cheongju 28644, Republic of Korea; brightdragom@gmail.com; 2 Human IT Convergence Research Center, Korea Electronics Technology Institute, Seongnam 13509, Republic of Korea; cjs97111@gmail.com; 3 School of Information and Communication Engineering, Chungbuk National University, Cheongju 28644, Republic of Korea; mchoi@chungbuk.ac.kr

**Keywords:** blockchain, consensus algorithm, PBFT, scalability, location-based clustering, dynamic hierarchy management

## Abstract

Blockchain is a distributed database technology that operates in a P2P network and is used in various domains. Depending on its structure, blockchain can be classified into types such as public and private. A consensus algorithm is essential in blockchain, and various consensus algorithms have been applied. In particular, a non-competitive consensus algorithm called PBFT is mainly used in private blockchains. However, there are limitations to scalability. This paper proposes an enhanced PBFT with dynamic hierarchy management and location-based clustering to overcome these problems. The proposed method clusters nodes based on location information and adjusts the dynamic hierarchy to optimize consensus latency. As a result of the experiment, the proposed PBFT showed significant performance improvement compared to the existing typical PBFT and Dynamic Layer Management PBFT (DLM-PBFT). The proposed PBFT method showed a processing performance improvement rate of approximately 107% to 128% compared to PBFT, and 11% to 99% compared to DLM-PBFT.

## 1. Introduction

Blockchain provides a high level of security as a distributed database technology [1,2]. Operating within a Peer-to-Peer (P2P) network, it involves nodes with various roles and purposes participating in the network [3]. Blockchain offers the advantage of storing transactions between nodes within each node’s distributed ledger, enhancing resistance to malicious attacks and making it nearly impervious to unauthorized alterations of stored transaction information [4]. It has been widely adopted in various cryptocurrencies, including Bitcoin, due to these security features [5]. Beyond cryptocurrencies, blockchain is utilized in diverse sectors like gaming, healthcare, smart factories, and ID cards. There is ongoing research to expand its applications to emerging domains, including metaverses and NFTs [3,6,7].

Blockchain can be broadly categorized into three types, depending on the purpose of the operating network and the roles of the participating nodes [5]. A public blockchain is an open blockchain where anyone can participate as a node in the blockchain network [8]. The nodes that participate in the network share the results with respect to the generated transactions. On the other hand, a private blockchain involves only authorized nodes from institutions and organizations. Therefore, private blockchains exhibit higher levels of trustworthiness compared to public blockchains [6]. However, opinions exist suggesting that this authorization by institutions and organizations actually undermines the decentralization of the blockchain. Lastly, there is the concept of a hybrid blockchain. A hybrid blockchain combines features of both public and private blockchains. It requires permission for significant transactions or cryptocurrencies, while making other data public. As a result, a hybrid blockchain can limit anonymity compared to private blockchains, but it maintains security through data anonymity. However, private blockchains require network access permissions to participate, which some argue compromises decentralization.

Blockchain employs consensus algorithms. This is intended to maintain an agreed-upon state among multiple participants in a distributed environment and to maintain consistent data. Consensus algorithms ensure network synchronization and reliability as numerous nodes simultaneously validate transaction information and generate new blocks. Each blockchain utilizes a consensus algorithm suited to its network to determine, store, and manage identical transactions. Among these, private blockchains restrict network participation to pre-authorized nodes and may adopt non-competitive consensus algorithms due to the absence of mining rewards.

PBFT (Practical Byzantine Fault Tolerance) is a prominent non-competitive consensus algorithm [9]. Employing a voting-based approach results in less wastage of computational resources compared to competitive consensus algorithms. With the participation of all network nodes in the consensus process, PBFT achieves a high degree of reliability, enabling consensus completion even in the presence of up to 33% malicious nodes. However, PBFT has a drawback in terms of scalability, as its time complexity when the number of nodes participating in the blockchain network is denoted as *n* is $O(n^{2})$ [5]. Scalability is a critical factor determining the performance and reliability of blockchain networks, especially in handling diverse and large volumes of transactions.

Consequently, many research endeavors are currently in progress to enhance both the reliability and scalability of PBFT [10,11,12,13]. To address the scalability issues of typical PBFT, there are several methods: forming smaller groups and conducting consensus within these groups, utilizing trust scores in PBFT where representative nodes with high trust scores participate in the consensus, and employing multi-layered structures to establish efficient blockchain networks. Group-based PBFT divides nodes into multiple groups, conducting PBFT within each group or forming a consensus group where a subset of the total nodes act as representatives to perform PBFT [14]. Trust score-based PBFT calculates the trust scores of each node and utilizes a method where nodes with high trust scores participate in the consensus as representatives, or consensus groups are formed based on trust scores when organizing groups [15,16]. Multi-layered PBFT divides the nodes participating in the blockchain into groups and forms a hierarchical structure for each group when conducting a consensus based on PBFT [13,17]. This approach facilitates faster consensus. Additionally, a method where nodes from a specific layer act as representatives to conduct the consensus can also be applied [17]. In previous research, when using the PBFT consensus algorithm, the approach often involved limiting the number of participating nodes to enhance consensus performance. However, this method can reduce the reliability of the consensus results, as it decreases the number of nodes verifying the block or the consensus message. Additionally, there should be more consideration for communication delays, such as the propagation delay time between participating nodes. The communication delay between nodes is a critical factor in PBFT-based consensus, particularly as the number of communication messages increases dramatically with the increasing number of participating nodes [16,18]. This can lead to performance degradation, especially in scenarios where grouped nodes are significantly distant from each other compared to those that are closer. Therefore, in PBFT-based blockchain consensus processes, research is needed on methods that ensure the participation of all nodes while minimizing communication delays when forming groups based on a hierarchical structure. Additionally, as the optimal hierarchical structure can vary depending on a blockchain network environment, such as the number of nodes participating in the blockchain, it is necessary to adjust the hierarchical structure dynamically.

This paper proposes an enhanced PBFT method via dynamic hierarchy management and location-based clustering to improve group-based PBFTs. In the proposed PBFT method, each node participating in the blockchain network is clustered based on their location, and groups are created for each cluster. Additionally, each cluster’s group is structured in a hierarchy, and consensus is carried out based on PBFT. Therefore, as nodes in close proximity are organized into clusters and groups, the communication delay time for sending and receiving consensus messages between each node can be minimized [19]. For this purpose, we propose algorithms for clustering all nodes and dynamic hierarchy management. Our method dynamically constructs a hierarchy structure by calculating an expected consensus time for each cluster to provide optimal performance in terms of consensus time.

The proposed method addresses latency issues by implementing a strategy of clustering nodes based on their location information, resulting in enhanced performance in consensus time. This improvement is attributed to adapting an optimal hierarchy structure tailored according to the number of nodes participating within each cluster. To validate the efficacy of the proposed scheme, a simulation study was conducted comparing the proposed PBFT scheme with the conventional PBFT and Dynamic Layer Management PBFT (DLM-PBFT) [20]. This study’s findings revealed a significant performance enhancement; specifically, there was a minimum improvement of approximately 107% with a network of 160 nodes and a maximum improvement of about 128% when the network comprised 300 nodes in terms of the consensus processing time. When compared to DLM-PBFT, the proposed scheme exhibited its minimal performance improvement with 120 nodes, showing an improvement rate of approximately 90% in terms of the consensus processing time.

The proposed PBFT method, with its innovative clustering and dynamic grouping, holds promise for diverse applications, particularly in sectors where rapid and scalable consensus is important. By minimizing communication latency and efficiently managing communication overhead, this method can revolutionize blockchain-based systems in finance, supply chain management, and beyond, offering enhanced security and reliability.

The structure of this paper is as follows. Section 2 provides an overview of relevant background knowledge and related research. Section 3 details the methodology of the proposed approach. Section 4 is dedicated to this proposed method’s performance evaluation and analysis. Section 5 describes the discussion on the results of the paper. Finally, Section 6 concludes the paper with a summary of the findings and potential directions for future research.

## 2. Related Works

### 2.1. PBFT

**Figure 1:**
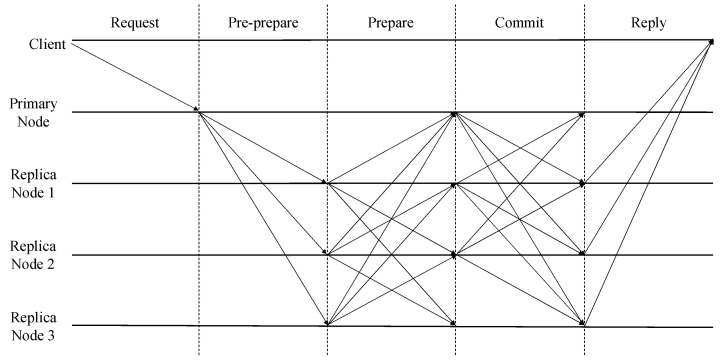
An example of the typical operation process of PBFT.

PBFT (Practical Byzantine Fault Tolerance) is a consensus algorithm designed to function in asynchronous networks. It guarantees consensus when there are more than 2f+1 nodes in total, even if *f* of them are malicious nodes (known as Byzantine nodes). This means the PBFT algorithm can achieve consensus even if up to 33% of the total nodes are malicious. PBFT is a representative non-competitive consensus algorithm used in private blockchains. Figure 1 illustrates the typical operation process of PBFT.

As depicted in Figure 1, when a new request is generated, the client sends a consensus request message to the primary node. Upon receiving this message, the primary node initiates the pre-prepare phase. The primary node then broadcasts the consensus request message to all other replica nodes participating in the consensus, excluding itself. The replica nodes, upon receiving the message from the primary node, validate it. If the message is deemed valid, the replica nodes broadcast prepare messages to the other nodes. Upon receiving 2f+1 prepare messages, each node transitions to the prepared state and initiates the commit phase. Nodes in the commit phase broadcast commit messages to other nodes. When a node receives 2f+1 commit messages, it transitions to the commit certificate state and updates its blockchain ledger.

During the execution of the consensus process, PBFT requires each node to broadcast messages twice to all other nodes, excluding itself, during both the prepare and commit phases to achieve consensus. Consequently, PBFT’s communication complexity is $O(n^{2})$ when there are *n* total nodes. This results in scalability challenges for PBFT as the number of nodes participating in the consensus grows [15,21].

### 2.2. SDMA-PBFT

SDMA-PBFT enhances the consensus performance of PBFT by dividing the participating nodes into smaller groups and establishing a hierarchical structure among these groups [17]. Initially, the client node broadcasts a consensus request message to the agent nodes. Each agent node acts as a leader for its corresponding lower-level group. Upon receiving the consensus request message, agent nodes broadcast it to any existing lower-level groups. If there are no additional lower-level groups to broadcast the consensus message, then those agent nodes initiate the PBFT consensus process within their group. Once consensus is achieved across the groups, the results are relayed back to the client node. Considering *n* consensus nodes overall and *k* nodes in each group, the communication complexity of SDMA-PBFT is calculated as $O(n\times k\times \log_{k}n)$. SDMA-PBFT ensures determinism by involving all nodes in the consensus and partitioning the entire set of nodes into smaller groups for PBFT-based consensus. However, since SDMA-PBFT employs a fixed value for *k*, which significantly impacts the consensus execution time, it may not consistently ensure optimal performance in terms of the total execution time required for each consensus operation, especially as the number of nodes in the consensus network varies.

### 2.3. G-PBFT

G-PBFT is a method designed for IoT-blockchain applications, taking advantage of the limited resources of IoT devices [22]. Most IoT devices are stationed in fixed locations for data collection and processing, and these stationary devices often outperform mobile IoT devices in terms of their capabilities, such as computing power. Compared to mobile IoT devices, stationary IoT devices possess greater computational capabilities. G-PBFT capitalizes on this characteristic by creating consensus groups based on the location information of the IoT devices. Moreover, the geographic attributes of these stationary devices are used to guard against Sybil attacks. The location information of specific IoT devices is embedded in the genesis block, and the nodes contained within are chosen as endorsers for the consensus process.

### 2.4. Dynamic Layer Management PBFT

The Dynamic Layer Management PBFT (DLM-PBFT) is proposed to address the scalability challenges of traditional PBFT, where the scalability decreases as the number of nodes increases [20]. The authors propose a model to estimate consensus time, taking into account the hierarchical structure and the overall number of nodes in the blockchain network. Based on the consensus time estimation model, DLM-PBFT dynamically adjusts the network’s hierarchical structure to enhance consensus performance. This allows for rapid consensus regardless of fluctuations in the number of nodes in the network. Experimental results demonstrate that DLM-PBFT offers enhanced processing speed compared to both traditional PBFT and SDMA-PBFT. However, DLM-PBFT did not consider the latency between each node, which is an important factor when forming groups and hierarchical structure.

The following Table 1 summarizes the differences between the existing methods and the proposed method. As summarized in Table 1, the proposed method has a lower time complexity in terms of message exchange frequency compared to existing methods. Additionally, it supports clustering, grouping, and dynamic hierarchy management to enhance the scalability of PBFT by reducing the consensus time.

As shown in Table 1, the communication complexity of the typical PBFT is $O(N^{2})$. As explained earlier, G-PBFT divides all nodes in the network into multiple groups based on their location information, and each group performs PBFT-based consensus. Therefore, if we represent the number of nodes within a single group as $N_{g_{node}}$ and the total number of groups as $N_{group}$, then the communication complexity is $O(N_{group}\times {N_{g_{node}}}^{2})$. The communication complexity of SDMA-PBFT can be described as follows. If we define the size of each area within SDMA-PBFT as *K* and the total number of nodes participating in the network as *N*, the number of areas is given by N/K and the number of hierarchical layers is represented by $\log_{K}N$. Consequently, the overall communication complexity becomes $O(N\times K\times \log_{K}N)$. In DLM-PBFT, given that the total number of nodes is denoted by *N* and the maximum number of nodes within a group is *K*, the number of groups can be represented as N/K. As a result, the communication complexity can be expressed as $O(N\times K^{2})$. The communication complexity of the proposed PBFT method is $O(N_{cluster}\times N_{node}\times N_{g\_node}^{2})$, which is further detailed in Section 3.3.3.

## 3. Proposed PBFT Architecture and Model

**Figure 2:**
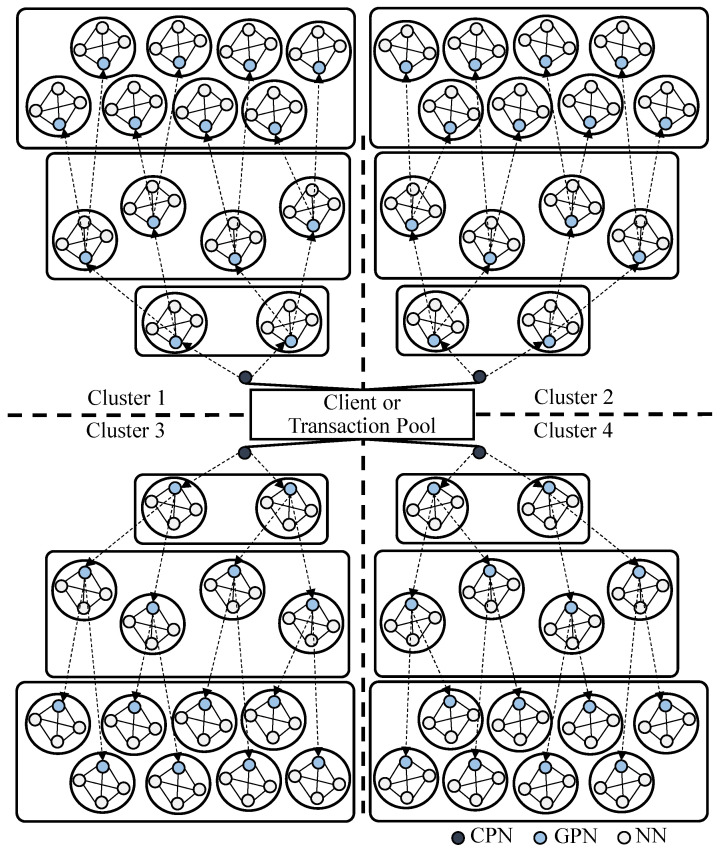
An example of proposed PBFT architecture.

This section describes the proposed enhanced PBFT via dynamic hierarchy management and location-based clustering. Our enhanced PBFT provides improved reliability and scalability compared to the DLM-PBFT and typical PBFTs. Figure 2 shows an example of the proposed PBFT architecture.

Due to blockchain networks’ Peer-to-Peer (P2P) communication nature, communication latency tends to increase as the distance between nodes grows. Minimizing communication latency is crucial for reducing the consensus time [23]. In the case of private blockchains, where node locations are usually static, this characteristic can be leveraged for efficient clustering. Our proposed method clusters nodes based on location information, aiming to benefit from reduced communication latency. Such clustering minimizes network communication latency and reduces the time required to reach consensus.

Furthermore, the proposed PBFT method enhances dynamic hierarchy management and grouping algorithms when compared to the DLM-PBFT. In other words, the proposed method can be applied to large-scale networks by utilizing a hierarchy formation algorithm based on location-based clustering [24].

In the rest of this section, we will explain the blockchain network structure where the proposed method is applied, the types of nodes that constitute this blockchain network, and the roles of each node. Additionally, we will explain how clustering is configured in the proposed method, and how we dynamically change the hierarchical structure within each cluster to reduce the time required for consensus.

### 3.1. Roles of Nodes and Network Architecture

The proposed method is designed to operate on a private blockchain, wherein institutions and operators manage the nodes and hierarchies. Due to the intrinsic nature of private blockchains, the locations of participating nodes tend to remain stable. Furthermore, gathering node information becomes straightforward since only verified nodes can participate. Capitalizing on these features, our method clusters all participating nodes based on their location data as shown in Figure 2, and establishes a hierarchical structure for the nodes within each cluster.

In the proposed PBFT, nodes participating in the blockchain network for consensus are categorized into three main roles:

- CPN (Cluster Primary Node): CPNs are responsible for managing the cluster.
- GPN (Group Primary Node): GPNs manage and lead consensus within the group.
- NN (Normal Node): NNs participate in consensus and verify consensus messages.

Detailed explanations of each node’s role can be found in Section 3.1.1, Section 3.1.2 and Section 3.1.3. Each node participating in the blockchain where the proposed method is applied performs one of the three roles described earlier.

During the consensus process, the CPN broadcasts the consensus message it receives from the client to the GPNs in the first layer of the cluster under its management. Subsequently, each GPN forwards the consensus message from the CPN to the NNs in its group and to the GPNs in its subgroup of the sub-layer. NNs within the group execute PBFT and participate in transaction verification under the management of the GPN. The role of each node is not fixed and can change during the repeated consensus process.

#### 3.1.1. CPN (Cluster Primary Node)

The Cluster Primary Node (CPN) is responsible for cluster management. The role includes managing and supervising groups and hierarchies within the cluster. The CPN initiates the consensus process by broadcasting a consensus message to the GPNs in the topmost layer. It then aggregates the results from each group, and then summarizes the consensus results, and sends them back to the client. CPNs directly participate in the consensus process and play an important role, CPNs must be chosen from nodes that are highly trustworthy. Additionally, the system may consist of multiple CPNs to handle various errors, such as system failures.

In the proposed PBFT method, GPNs are randomly selected for consensus during the initial layering and grouping stages. Subsequently, groups are made by choosing NNs to participate in PBFT with the selected GPNs. When the consensus operation begins, the CPN broadcasts the consensus messages to the GPNs in its managed cluster’s topmost layer (Layer 1). These consensus messages originate from a client’s or manager’s request, pulling transactions from the transaction pool to create a block message. To explain the roles performed by the CPNs in more detail, it is as follows.

After broadcasting, the CPN waits for each group to reach a consensus on the messages. Once the CPN receives the consensus results from the GPNs during the Pre-Reply phase, it aggregates these results and relays the overall consensus of the cluster to the client or manager. Throughout the consensus process, the CPN monitors changes in the number of nodes in the cluster and counts the number of consensus operations performed. If there is a change in the number of participating nodes or consensus operations exceed a predetermined threshold, the CPN dynamically re-adjusts the hierarchical structure and grouping. Figure 2 illustrates a blockchain network utilizing the proposed PBFT method. In this figure, black nodes represent CPNs, and each cluster contains one CPN.

#### 3.1.2. GPN (Group Primary Node)

The GPN is the representative node responsible for group management within the cluster selected by the CPN. The GPN broadcasts the consensus message received from the CPN to the lower layer for consensus. It plays an important role in achieving consensus within the group. In summary, the GPN manages the consensus process within the group it manages and aggregates the consensus results from within the group. To explain the role of the GPN in more detail, it is as follows.

After receiving the consensus message from the CPN, if there is a GPN in the lower layer, the GPN broadcasts the received consensus message to the GPNs in the lower layer. If there is no GPN to forward the consensus message to a lower layer, each GPN performs the PBFT-based consensus algorithm with the NNs within its group. In this process, the GPN is responsible for achieving consensus within the group and serves as the primary node of the typical PBFT protocol. Once the consensus within the group is complete, the GPN aggregates the consensus results and returns them to the CPN.

As we described, GPNs play an important role in starting and finishing the consensus algorithm in each group. Therefore, problems may occur if the GPN behaves abnormally due to network errors, malicious activity, or other reasons. To mitigate these risks, we can leverage the nature of private blockchains to select GPNs based on trusted nodes. Additionally, institutions and operators can impose severe penalties if abnormal behavior occurs, including long-term cancellation of GPN status.

#### 3.1.3. NN (Normal Node)

An NN is typically a regular node participating in consensus. It belongs to a specific group managed by a certain GPN and performs verification on the consensus messages delivered by the GPN. Under the management of the GPN, the NN executes the PBFT-based consensus algorithm. During the Commit phase, each NN temporarily incorporates the completed block into its chain or ledger if there are no abnormalities with the consensus message. For trustworthiness and fairness within the blockchain network, if an NN is selected as a GPN, it can play the role of a GPN.

### 3.2. Network Architecture

The proposed PBFT method is designed to perform location-based clustering for nodes participating in the network and dynamically adjust the hierarchical structure of the blockchain network. Figure 2 illustrates an example of the blockchain network structure proposed in this paper. In Figure 2, 224 nodes participating in the network are evenly distributed in each cluster. Each cluster is divided and managed by 56 nodes. Each cluster has three hierarchical layers, with one CPN managing each cluster. Furthermore, each group comprises one GPN and three NNs, representing a blockchain network structure. The maximum number of child groups is 2.

A distinctive feature of the proposed PBFT method is that it dynamically changes the network hierarchy and group structure, considering factors such as the number of nodes participating in the network and latencies. Therefore, the maximum number of child nodes and the hierarchical structure of the network can vary based on the estimated consensus time estimation model in Section 3.3.2.

The location-based clustering can be applied to the proposed PBFT method in various ways. For example, in the proposed PBFT method, clustering can be applied based on countries or cities because it is easy to determine the location of nodes participating in consensus within a private blockchain system. The proposed PBFT method utilizes these characteristics to divide and manage all nodes into multiple clusters to minimize communication delay during the consensus process. This can bring benefits in terms of consensus time. Detailed results on comparing consensus time are described in the experimental results.

### 3.3. Consensus Process of Proposed PBFT

The consensus process of the PBFT method proposed in this paper is broadly divided into three stages: clustering, grouping, and the main consensus. Clustering and grouping are introduced to reduce communication delays among nodes within the blockchain network, thus ensuring enhanced scalability in comparison to the conventional PBFT and DLM-PBFT methods. A meticulous design approach is necessary to minimize the overhead of these additional clustering and grouping stages. Table 2 shows the notations and descriptions used in this paper to explain the proposed PBFT method.

#### 3.3.1. Clustering Stage

PBFT suffers from scalability issues as the number of nodes in a blockchain network increases [14]. The proposed PBFT addresses these issues by implementing a layered network structure that utilizes location-based clustering and dynamic group management. Location-based clustering is effective in reducing communication latency within blockchain networks. Generally, the propagation delay increases as the distance between two communicating nodes increases. Therefore, the overall communication latency increases.

**Figure 3:**
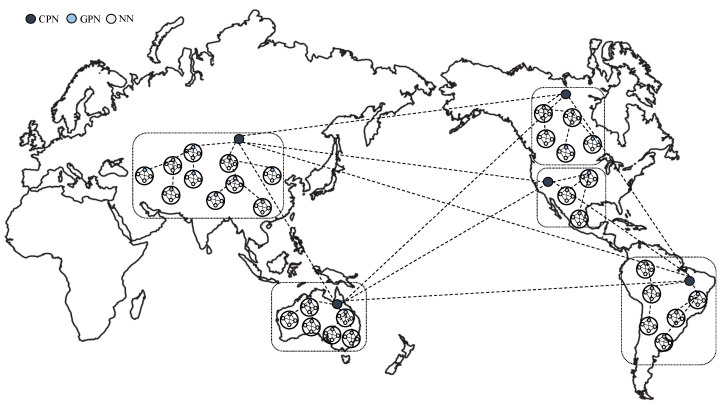
An example of blockchain network structure with location-based clustering.

Proper clustering and grouping management of network participant nodes is crucial for minimizing communication delays. When clustering, it is possible to manage nodes at the national or city levels. Clustering nodes at the city level in the blockchain network would result in an increased number of CPNs managing each cluster. In such cases, broadcasting consensus messages and aggregating results can lead to considerable overhead. In contrast, clustering at the national level can reduce the potential overhead for CPNs, given the relatively smaller number of clusters involved. However, this approach may result in the formation of multiple groups within each cluster, increasing the consensus time for each group. Consequently, achieving a good balance between the total number of clusters and groups is crucial. Figure 3 illustrates a blockchain network structured based on continental locations, comprising five clusters. Each CPN and GPN can manage up to two subgroups within the example structure, as shown in Figure 2.

As shown in Figure 3, the consensus process based on PBFT is conducted with these groups as the fundamental units by utilizing the nodes within each cluster to form groups. Such clustering ensures that relatively distant nodes do not exchange messages during the consensus process. Consequently, this approach can significantly reduce the time required for achieving consensus.

The proposed PBFT method ensures that nodes other than the CPN of each cluster cannot communicate with nodes from different clusters during the consensus process. In cases where the number of participating nodes in terms of countries or cities is very small, nodes can be evenly distributed among multiple clusters based on their physical locations. However, a significant disparity in the number of nodes among specific clusters may lead to substantial differences in the time required for consensus within each cluster. During the clustering stage, it is essential to cluster nodes participating in the blockchain network based on their location information so that each cluster has an evenly distributed number of nodes as much as possible.

#### 3.3.2. Consensus Stage

**Figure 4:**
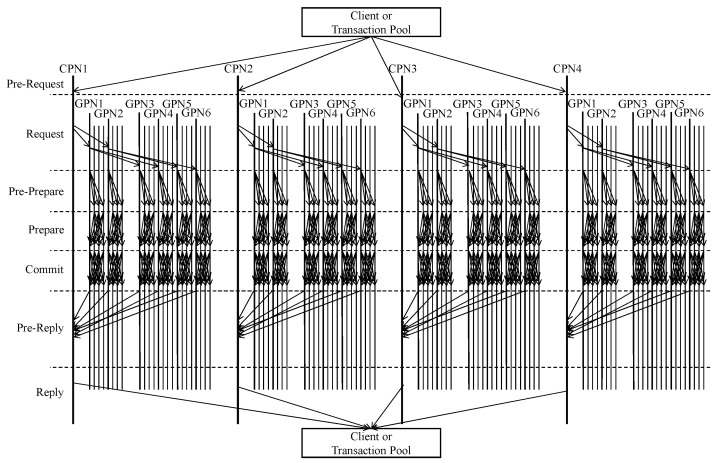
An example of consensus process of proposed PBFT.

The proposed PBFT method divides all nodes participating in the network into several clusters. Nodes within each cluster are further divided into groups for the consensus process. Consensus times tend to increase as the number of nodes in a cluster increases. To solve this problem, we improved and applied dynamic grouping and hierarchy management methods. Figure 4 shows the consensus process of the proposed PBFT method. As shown in Figure 4, the consensus process of the proposed PBFT method is subdivided into seven phases.

The consensus algorithm for each cluster comprises the following steps:

- Pre-Request Phase: This involves sending a consensus request message to each cluster.
- Request Phase: The Cluster Primary Node (CPN) verifies the received message and broadcasts it to the lower-level Group Primary Nodes (GPNs). Also, the GPN that receives the message propagates the message to GPNs in its lower layers.
- Pre-Prepare phase: The GPN of each group forwards the request message to the NNs of its own group and performs verification.
- Prepare Phase: Each group prepares for consensus by verifying the message and its intent.
- Commit Phase: Consensus is confirmed within each group, and the decision to commit the transaction is made.
- Pre-Reply Phase: This phase aggregates the consensus results from each group within the cluster.
- Reply Phase: The final consensus results are returned to the client.

Furthermore, we propose a model to estimate the expected consensus time of these detailed phases. We also use the expected consensus time estimation model to find the optimal hierarchy structure. In other words, the proposed PBFT dynamically adjusts the number of layers and the number of nodes per group, which can minimize consensus time using the expected consensus time estimation model. The following is a detailed description of the actions taken in each phase and a comprehensive explanation of the execution time estimation model for each phase.

(1) Pre-Request Phase: This is the phase where the client or transaction pool manager logically converts the transaction to be agreed upon into block format and creates a consensus message. The consensus message generated in this way is sent to the CPN of each cluster. In this step, the consensus message is assigned a unique number. The previous unique number is reassigned if consensus on the previous consensus message fails and is not reflected in the blockchain. This mechanism allows information to be passed on to nodes participating in the blockchain without a separate synchronization step or reflection confirmation.

After receiving the consensus message, the CPN verifies its contents and ensures there are no problems. During this verification, the CPN checks the unique identifier of the consensus message. If it contains information indicating that changes to the ledger are required based on the cluster’s previous consensus, the CPN initiates the transition to the next block. The estimated time required for this Pre-Request phase can be calculated using the Equation (1).

(1) $$T_{Pre\text{-}Request}=\left(\frac{S_{block}}{BW}+L_{inter}\right)\times N_{cluster}$$

(2) Request Phase: This phase marks the initiation of consensus for each cluster. The CPN evaluates the integrity of the consensus messages received from the client or administrator. In the absence of anomalies, the CPN broadcasts the consensus message to the GPNs located in the first layer of the hierarchy of the cluster it manages. At this point, the CPN checks for any changes in the number of nodes within the blockchain network of the cluster it manages. If changes are detected, it proceeds with the reconfiguration of groups and the hierarchical structure. The process of group reconfiguration and the transmission of the reconfigured group information is explained in detail in Section 3.3.4.

Upon receiving the message, a GPN (Group Primary Node) passes it down to another GPN in its lower layer if one exists. If no additional lower-layer GPNs are available, the GPN transitions to the Pre-Prepare phase. The estimated time required for the Request phase is calculated using the formula provided in Equation (2).

(2) $$T_{Request}=\left(\frac{N_{child}\times S_{block}}{BW}+L_{intra}\right)\times N_{hierachy}$$

As shown in Equation (2), the equation calculates the time it takes for a message to be sent from a CPN or GPN node to all its child GPN nodes and then multiplies it by the number of layers in the hierarchy that this process needs to happen. This gives an overall estimate of the time required for the Request phase of the consensus process.

(3) Pre-Prepare Phase: This phase marks the initiation of PBFT within each group. The GPN first assesses the integrity of the consensus message it receives from the Cluster Primary Node (CPN). This is a crucial step to ensure that the message has not been tampered with and is legitimate. If the consensus message is intact and free from anomalies, the GPN adds its signature to it. This signature acts as a stamp of approval from the GPN, indicating that the message has passed its checks. After signing the consensus message, the GPN broadcasts the consensus message that needs to be agreed upon to all Normal Nodes (NNs) within its group. This broadcasting is a form of communication where the message is sent out to all nodes in each group without explicitly targeting any single node. The equation for estimating the time required for the Pre-Prepare phase is given by Equation (3)

(3) $$T_{Pre\text{-}Prepare}=\frac{\left(N_{g\_node}-1\right)\times S_{block}}{BW}+L_{intra}$$

(4) Prepare Phase: This phase serves as the initial verification step in the main consensus process. Upon receiving the consensus message from the GPN, each Normal Node (NN) validates its contents. This involves checking for any issues with the message itself as well as verifying the signatures of the CPN and GPN. If the message and signatures are valid, the NN adds its own signature and broadcasts Prepare messages to other nodes within its group. However, if the consensus message is incorrectly delivered to nodes that are not receiving the group information properly, in that case, the NN returns the current group information, indicating that a change has occurred.

In the Prepare phase, a Normal Node (NN) transitions to the Prepared state when it receives more than 2f+1 Prepare messages. This count must be concerning the total number of nodes in the group, denoted by $N_{g\_node}$, where *f* represents the number of malicious nodes in the group. Upon reaching this threshold, the NN proceeds to the subsequent Commit phase. The estimated time required for the Prepare phase is calculated using Equation (4).

(4) $$T_{Prepare}=\left(\frac{\left(N_{g\_node}-1\right)\times S_{block}}{BW}+L_{intra}\right)+T_{verify}$$

(5) Commit Phase: This phase is the second verification step. Nodes within the group that have transitioned to the Prepared state broadcast Commit messages to the members of their respective groups. If the GPN and NNs receive more than 2f+1 Commit messages for their group, and the total number of group nodes is $N_{g\_node}$, they change their state to Committed. They then temporarily reflect the block in their blockchain or ledger.

Suppose the unique identification number of the next consensus block differs from the temporarily stored block. In that case, it is considered that the previous block has been successfully integrated into the entire blockchain network. Once the integration of the previous block is confirmed, each node officially incorporates the temporarily stored block into its own blockchain or ledger. The estimated time required for the fifth Commit phase is calculated by Equation (5).

(5) $$T_{Commit}=\left(\frac{\left(N_{g\_node}-1\right)\times S_{block}}{BW}+L_{intra}\right)+T_{setBlock}$$

(6) Pre-Reply Phase: In this phase, the consensus results of groups within the cluster are aggregated. All Group Primary Nodes (GPNs) that have received more than 2f+1 Commit messages forward the consensus results of each group to their respective Cluster Primary Nodes (CPNs). The CPN considers the block valid and temporarily records it on the blockchain or ledger when it receives approval from over 50% of the total groups within the cluster.

If we assume that all GPNs of each group sequentially transmit the inter-group consensus results, then the estimated time required for the CPN to complete the collection of intra-cluster consensus results is given in Equation (6).

(6) $$T_{Pre\text{-}Reply}=\frac{\left(N_{node}/N_{g\_node}\right)/2\times S_{Reply}}{BW}+L_{intra}$$

(7) Reply Phase: The Reply phase is the final step, where the consensus results of each cluster are finally aggregated. Each cluster’s consensus result is transmitted to the client via the CPN, indicating whether the consensus message has been recorded on the ledger for each cluster. The client updates the blockchain or ledger only if a consensus is reached, with $f_{total}$ or more of the participating network nodes in agreement. $f_{total}$ represents the minimum number of nodes required for the consensus. If the consensus is not achieved, the transaction details from the message may be deferred for inclusion in subsequent blocks during the consensus process for eventual recording on the ledger. The estimated time required for the Reply phase is provided in Equation (7).

(7) $$T_{Reply}=\left(\frac{S_{Reply}}{BW}+L_{inter}\right)\times N_{cluster}$$

We have provided a detailed explanation of the seven phases that constitute the consensus process of the proposed PBFT method. In addition, we have described models that can calculate the expected execution time for each phase. In the consensus process of the proposed PBFT method, the total expected consensus time, denoted as Equation (8), can be calculated.

As shown in Equation (8), the total expected consensus time is the summation of all Equations (1)–(7). The time required for consensus varies depending on the total number of layers in the blockchain network, the number of nodes in each group, and the maximum number of child nodes a GPN can have. Therefore, in the proposed PBFT method, Equation (8) can be utilized to roughly estimate the time required for consensus, taking into account various blockchain network configurations. This calculated expected consensus time can be used to dynamically change the grouping and hierarchical structure when the number of nodes participating in the cluster changes or when there is a change in the hierarchical structure.

(8) $$\begin{array}{c} \;T_{total}=T_{Pre\text{-}Request}+T_{Request}+T_{Pre\text{-}Prepare}+T_{Prepare}+T_{Commit}+T_{Pre\text{-}Reply}+T_{Reply} \end{array}$$

#### 3.3.3. Communication Complexity Analysis

Two assumptions were made to compute the communication complexity of the proposed PBFT method. The first is that all clusters comprise an identical number of nodes. The second is that the hierarchical structure of each cluster forms a perfect tree. During the Request phase, each Cluster Primary Node (CPN) broadcasts consensus messages to their respective Group Primary Nodes (GPNs). Upon receiving these messages, the GPNs then relay the consensus messages down to the lower-level GPNs in their hierarchical network structure. This process is repeated according to the number of groups present in each cluster. The total number of groups within a single cluster is defined by Equation (9). Consequently, as the Request phase is conducted for each cluster, its communication complexity can be described by Equation (11). In the Pre-Prepare phase, each group’s Group Primary Node (GPN) broadcasts consensus messages to the Normal Nodes (NNs) within its group. In this phase, $N_{g\_node}-1$ communications occur for each group. Therefore, the total number of communications for the Pre-Prepare phase is represented by Equation (12). The Prepare and Commit phases serve as consensus verification processes. Within each group, ${\left(N_{g\_node}-1\right)}^{2}$ communications take place. Consequently, the communication complexity for these phases is outlined by Equation (13). In the Pre-Reply phase, each group’s GPN returns the consensus results to its corresponding CPN. Thus, communication occurs as frequently as the number of groups, as denoted by Equation (14). The final Reply phase involves delivering the consensus results of each cluster to the client, with communication occurring for each cluster as shown in Equation (15). Hence, the overall communication complexity of the proposed PBFT method can be represented by Equation (16).

(9) $$\mathrm{The}\;\mathrm{number}\;\mathrm{of}\;\mathrm{groups}\;\mathrm{in}\;\mathrm{each}\;\mathrm{cluster}=\left\lceil \frac{N_{node}}{N_{g\_node}}\right\rceil$$

(10) $$\left(\mathrm{Pre}\text{-}\mathrm{Request}\;\mathrm{Phase}\right)\;N_{cluster}$$

(11) $$\left(\mathrm{Request}\;\mathrm{Phase}\right)\;\left(\frac{N_{node}}{N_{g\_node}}\right)\times N_{cluster}$$

(12) $$\left(\mathrm{Pre}\text{-}\mathrm{Prepare}\;\mathrm{Phase}\right)\;\left(\left(N_{g\_node}-1\right)\times \frac{N_{node}}{N_{g\_node}}\right)\times N_{cluster}$$

(13) $$\left(\mathrm{Prepareand}\;\mathrm{and}\;\mathrm{Commit}\;\mathrm{Phase}\right)\;\left(\frac{N_{node}}{N_{g\_node}}\right)\times {\left(N_{g\_node}-1\right)}^{2}\times N_{cluster}$$

(14) $$\left(\mathrm{Pre}\text{-}\mathrm{Reply}\;\mathrm{Phase}\right)\;\left(\frac{N_{node}}{N_{g\_node}}\right)\times N_{cluster}$$

(15) $$\left(\mathrm{Reply}\;\mathrm{Phase}\right)\;N_{cluster}$$

(16) $$\left(\mathrm{Communication}\;\mathrm{Complex}\right)\;O\left(N_{cluster}\times N_{node}\times N_{g\_node}^{2}\right)$$

#### 3.3.4. Dynamic Group and Hierarchy Management

This section explains how the proposed PBFT dynamically changes and manages the number of groups and hierarchical structure. The expected consensus time for each cluster varies according to the hierarchical structure configuration. We can define a function in the form of $T_{total}\left(c,g,n\right)$ based on Equation (8). Here, *c* represents the maximum number of child nodes that both CPN and GPN can have, *g* denotes the number of nodes composing the group, and *n* denotes the total number of nodes within one cluster. For example, in the case of $T_{total}\left(4,4,120\right)$, where the maximum number of child nodes for both CPN and GPN is four, and four nodes form a group and 120 nodes in a single cluster, it calculates the expected time required for consensus. Therefore, using Equation (17), we can determine the best hierarchical structure and estimate the expected consensus time.

(17) $$T_{expected}=\min_{c,g,n}T_{total}\left(c,g,n\right)$$

In the proposed PBFT method, we designed an algorithm to dynamically adjust the hierarchical structure for each cluster based on Equation (17). Each CPN periodically monitors changes in the number of nodes participating in the blockchain network. Suppose there is a change in the number of participating nodes, or consensus has been reached more than a certain number of times after restructuring the network’s hierarchical structure. In that case, the CPN executes the algorithm to dynamically change the network’s hierarchical structure. As previously explained, each CPN uses Equation (17) to derive the best network hierarchical structure.

CPN includes information about the new groups and hierarchical structure in the consensus message during the Request phase and transmits it. Upon receiving the message containing group information, GPN carries out the consensus with the new group members. Algorithms 1 and 2 represent our dynamic group and hierarchy management algorithm used in the proposed PBFT method in pseudocode form.
**Algorithm 1** Check_Status()1:${\mathrm{TH}}_{\mathrm{consensus}}\leftarrow$ Set the threshold for the number of consensus processes that can be executed after reorganizing the network hierarchical structure2: $N_{\mathrm{consensus}}\leftarrow 0$3: **while** 1 **do**4:     Do consensus operation5:     $N_{\mathrm{consensus}}\leftarrow$**$N_{consensus}$** + 16:     **if** $N_{node}$ is changed? or $N_{consensus}>TH_{consensus}$ **then**7:         GHI← Call the **Get_Best_GHI()**8:        $N_{consensus}\leftarrow 0$9:        Propagate the GHI information to other nodes in the network10:    **end if**11:**end while**

Each CPN performs Algorithm 1. As depicted in Algorithm 1, it operates in a continuous loop to manage consensus operations within a blockchain network. Initially, it set a threshold value $TH_{consensus}$, which means the number of consensus processes permissible after restructuring the network’s hierarchical organization. The algorithm then initializes a counter, $N_{consensus}$, to zero. This counter tracks the number of consensus operations executed. As the algorithm enters its continuous loop, it undertakes a consensus operation and subsequently increments the $N_{consensus}$ counter. If either the number of nodes ($N_{node}$) has changed or the $N_{consensus}$ exceeds the predetermined $TH_{consensus}$ threshold, the algorithm calls the Get_Best_GHI() function. This function’s role is to procure the network’s best Grouping and Hierarchical Information (GHI). After obtaining the GHI, the counter $N_{consensus}$ is reset to zero. Then, it propagates the obtained GHI to other nodes within the network. This looped process ensures that the CPN constantly monitors and updates its hierarchical structure based on the frequency of consensus operations and any changes in participating nodes.
**Algorithm 2** Get_Best_GHI()1:$L_{\mathrm{inter}}\leftarrow$ Update the inter-cluster communication latency2:$L_{\mathrm{intra}}\leftarrow$ Update the intra-cluster communication latency3:BW← Update the average network bandwidth of nodes participating in the blockchain network4:$N_{\mathrm{node}}\leftarrow$ Update the total number of nodes participating in consensus within the cluster5:**Find** $GHI_{best}$ based on Equation (17) with $L_{\mathrm{inter}}$, $L_{\mathrm{intra}}$, BW6:**return** $GHI_{best}$

In Algorithm 2, it starts by updating the $L_{inter}$ value, which represents the communication latency between different clusters in the network. Subsequently, it updates the $L_{intra}$ value, which means the communication latency within a single cluster. The next step is to update the bandwidth parameter BW. This represents the average network bandwidth of the nodes that are participating in the blockchain network. The algorithm then updates the $N_{node}$ value, which means the total number of nodes participating in the consensus process within a particular cluster. The algorithm calculates $GHI_{best}$ with these updated values by utilizing Equation (17). This equation takes into account the inter-cluster latency ($L_{inter}$), intra-cluster latency ($L_{intra}$), and the average bandwidth (BW) to determine the best group and hierarchical configuration for the blockchain network. Finally, the algorithm returns the $GHI_{best}$ information, indicating the best grouping and hierarchical structure based on the current network conditions and the specified equation.

## 4. Performance Evaluations

### 4.1. Experiment Environment

Table 3 describes the computing environment utilized for the experiments. The experiments were conducted on a computer operating on the Ubuntu 20.04 LTS system. This system comprises a total memory capacity of 64 GB, distributed across four DDR4 16 GB modules. The proposed PBFT method was implemented using Python 3.7.13, and the aiohttp 3.8.1 Python library was employed for HTTP communication.

In order to compare the performance of the proposed PBFT method, the experiment compared the typical PBFT, DLM-PBFT, and the proposed PBFT method.

- Typical PBFT: this method is a kind of the traditional Practical Byzantine Fault Tolerance (PBFT), which is a consensus algorithm that allows a system to continue operation even if some nodes fail or act maliciously. It requires a series of communication steps among nodes to reach a consensus and guarantees system reliability. When the number of nodes participating in the blockchain is denoted as *N*, the communication complexity of this method is $O(N^{2})$.
- DLM-PBFT: it dynamically adjusts the network’s hierarchical structure to enhance consensus performance. However, in a single group, if a node with comparatively high latency is included, it can result in a degradation in the consensus performance.
- Proposed PBFT: the proposed PBFT method initially clusters the nodes participating in the blockchain network according to their location information. It then dynamically adjusts and manages the network’s hierarchical structure in response to the current network environment. Consequently, nodes with comparatively low mutual latency can group. Therefore, there is a further decrease in the consensus time.

The parameters used for the experiment are detailed in Table 4. To assess the effectiveness of the proposed PBFT method, experiments were conducted with node counts of 120, 160, 200, 240, 280, and 300. The maximum number of subgroups ($N_{child}$ for CPNs and GPNs) started at a minimum of two and increased incrementally in steps of two, reaching up to twenty. The number of nodes in a single group ($N_{g\_node}$) was consistently set at four. The network consists of four distinct clusters. Two types of communication latencies were used to simulate a blockchain network with distributed nodes: the latency within each cluster and the latency between clusters involving CPNs. The latency values were based on interactions within a Google Cloud region, reflecting an average of 126 ms between Los Angeles, USA, and Seoul, Korea. The intra-cluster latency was set at an average delay of 0.03 s for transmitting a 1 MB message. Moreover, experiments varied the communication delay ratio between $L_{inter}$ and $L_{intra}$, including ratios of 1:2, 1:4, and 1:8. In our experiments, we measured the cumulative time required to process a 1 MB message four times, and the comparison results are detailed in Section 4.2.

### 4.2. Experiment Results

#### 4.2.1. Consensus Message Processing Performance According to the Total Number of Nodes

**Figure 5:**
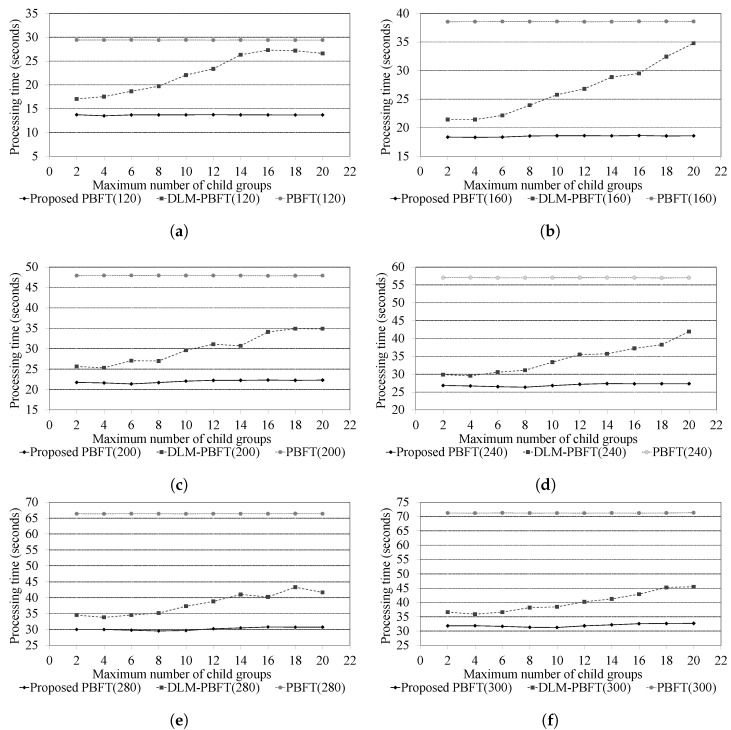
Results of the consensus message processing performance. (**a**) Total number of nodes ($N_{total}$) is 120. (**b**) Total number of nodes ($N_{total}$) is 160. (**c**) Total number of nodes ($N_{total}$) is 200. (**d**) Total number of nodes ($N_{total}$) is 240. (**e**) Total number of nodes ($N_{total}$) is 280. (**f**) Total number of nodes ($N_{total}$) is 300.

Figure 5 presents the consensus message processing performance based on the total number of nodes in the blockchain network. As depicted in Figure 5, the message processing performance of the proposed PBFT method, the typical PBFT, and the DLM-PBFT was compared, taking into account the maximum number of child groups in scenarios where the total number of nodes varies from 120 to 300. The experimental results indicated that the proposed method exhibited the best consensus message processing performance in comparison to both the DLM-PBFT and the typical PBFT. Both the DLM-PBFT and the proposed PBFT method surpass the performance of the typical PBFT. These two methods ensured effective consensus, even as the number of nodes expanded.

As illustrated in Figure 5, our proposed PBFT method demonstrated superior performance compared to DLM-PBFT. A key distinction between the DLM-PBFT and the proposed PBFT method is the absence of location-based clustering in DLM-PBFT. In the case of DLM-PBFT, the consensus time increases as the number of nodes in a cluster grows. This is because nodes with relatively longer communication delay times can be included when each group is formed. Thus, the location-based clustering feature of the proposed PBFT method gives it an advantage over DLM-PBFT in terms of the consensus processing time.

In Figure 5, it is evident that DLM-PBFT’s processing performance declined as the maximum number of child groups increased. However, when the maximum number of child groups was limited, such as 2 or 4, the performance difference was relatively minimal compared to other cases. As we explained, DLM-PBFT employs a group-based hierarchy structure, which offers some scalability as the number of nodes grows. With an increase in the maximum number of child groups, this hierarchical structure becomes less distinct and more akin to the typical PBFT structure. As a result, when the hierarchical structure can effectively utilize a smaller number of child groups, DLM-PBFT showed relatively good performance. But as this number grows, performance increasingly resembles that of PBFT.

As a result of the experiment, the proposed PBFT method showed an improvement in processing performance ranging from about 107% to about 128% compared to PBFT. And the proposed PBFT method showed an improvement in processing performance ranging from about 11% to about 99% compared to DLM-PBFT.

#### 4.2.2. Performance Results of the Proposed PBFT Method Based on the Number of Child Groups

**Figure 6:**
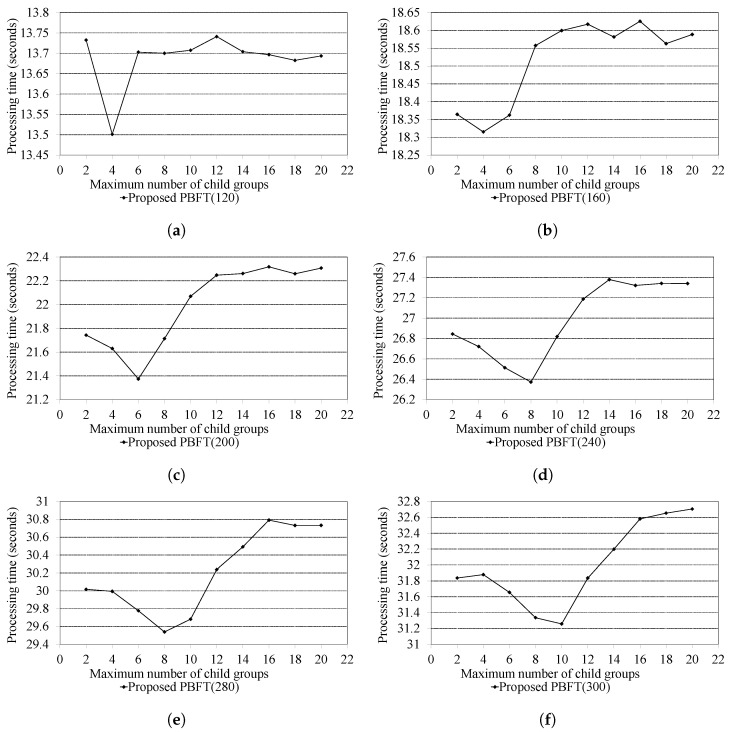
Results according to the maximum number of child groups of the proposed PBFT. (**a**) Total number of nodes ($N_{total}$) is 120. (**b**) Total number of nodes ($N_{total}$) is 160. (**c**) Total number of nodes ($N_{total}$) is 200. (**d**) Total number of nodes ($N_{total}$) is 240. (**e**) Total number of nodes ($N_{total}$) is 280. (**f**) Total number of nodes ($N_{total}$) is 300.

Figure 6 presents the consensus message processing time of the proposed PBFT method, varying with the number of child groups, as the total number of participating nodes in the blockchain changes. In the experiments, we varied the maximum number of child groups from 2 to 20 and assessed the total processing time across different node counts. Our observations indicated that the ideal number of child groups shifted as the total number of nodes increased. For configurations with 120 and 160 nodes, peak performance was obtained with four child groups during the formation of the hierarchical group structure. For the setup with 200 nodes, using six child groups resulted in quicker consensus processing than with four child groups. In situations with 240 and 280 nodes, the best performance was reached with eight child groups. For the 300 nodes, the best performance was recorded with 10 child groups.

As we explained, the experimental results indicate that the most efficient number of maximum child groups, providing the fastest processing performance, varies according to the number of nodes participating in the blockchain network. This means that there is a need for dynamic adjustments in the hierarchical structure based on the total number of nodes, rather than just using the fixed maximum number of child groups to a specific value. Additionally, it was observed that as the total depth of the hierarchical structure approached one layer, efficiency decreased in terms of the processing time. When there were 120 nodes, one cluster consisted of 30 nodes. In this scenario, the total number of layers of the hierarchical structure was one when the maximum number of child groups was set to eight. For 160 nodes, one cluster contained 40 nodes, and the total layer of the hierarchical structure was one when the maximum number of child groups was set to ten. As the node count increased to 200, the total layer of the hierarchical structure had one when the maximum number of child groups was fourteen. Similarly, for 240 nodes, it was 16 child groups. For 280 and 300 nodes, it was 20 child groups.

In short, as the hierarchical structure gradually disappears, performance decreases. This happens because as the hierarchical depth converges to one, each cluster performs consensus operations more similar to the typical PBFT. Therefore, it is crucial to establish an appropriate hierarchical structure that depends on the number of nodes participating in consensus after location-based clustering to achieve the best performance.

#### 4.2.3. Performance Results According to the Ratio of Intra to Inter Latency

**Figure 7:**
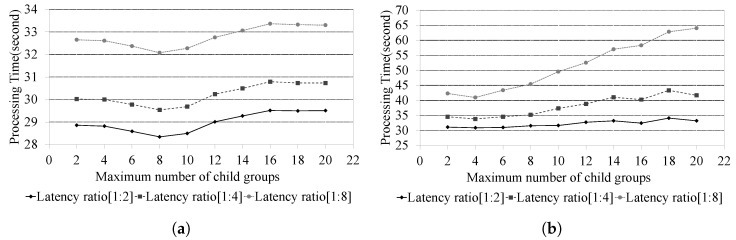
Results according to the ratio of intra to inter latency (Where, the total number of nodes ($N_{total}$) is 280). (**a**) Proposed PBFT. (**b**) DLM-PBFT.

In Section 4.2.3, we focus on experiments designed to study the performance effects associated with varying ratios of intra to inter latency. The primary difference between the proposed PBFT method and DLM-PBFT is whether location-based clustering is applied or not. Consequently, as the ratio of intra to inter latency increases, the proposed PBFT method is expected to outperform DLM-PBFT. Figure 7 presents the performance results for both the proposed PBFT method and DLM-PBFT, according to the ratio of inter latency to intra latency, where the total number of nodes ($N_{total}$) is 280. Experiments were conducted with intra to inter latency ratios set at 1:2, 1:4, and 1:8, respectively. In the experiments, the intra latency was set to constant at 0.03 s, while the inter latency was set to 0.063 s for a 1:2 ratio, 0.126 s for a 1:4 ratio, and 0.252 s for a 1:8 ratio.

Numerous communications occur during the consensus process in a blockchain network that operates based on P2P communication without relying on third parties or central institutions. Therefore, the latency incurred in each communication affects the overall consensus processing time. The experimental results depicted in Figure 7a,b demonstrate that the overall consensus processing time also increases as inter-latency increases.

However, when comparing scenarios with identical delay ratios in Figure 7a,b, the proposed PBFT method outperformed DLM-PBFT in consensus message processing time. This means the proposed method’s location-based clustering reduces the time each group needs for consensus, offering superior performance over DLM-PBFT. Furthermore, the proposed PBFT method consistently demonstrated stable performance despite continuous changes in the delay ratio. In contrast, DLM-PBFT exhibited performance degradation as the delay ratio increased.

## 5. Discussions

The proposed PBFT method performs clustering based on node location information to minimize the latency in sending and receiving consensus messages. Additionally, each cluster divides its nodes into groups and conducts consensus based on a hierarchical structure. The proposed method also dynamically changes the hierarchical structure in response to changes in the blockchain network. Based on these mechanisms, the proposed PBFT method can improve the scalability issues of the PBFT-based consensus algorithm by reducing the consensus time. However, it is necessary to discuss the following factors.

The Cluster Primary Node (CPN) is responsible for cluster management, which includes managing and supervising groups and hierarchies within the cluster. If the CPN is attacked, shut down, or compromised, the proposed PBFT scheme may not function properly. Our proposed scheme is intended for use in a relatively secure private blockchain network, where an administrator can preemptively block the participation of malicious nodes. However, the CPN may face operational issues due to network communication failures or physical factors. Therefore, when selecting CPNs, we can choose them based on their trustworthiness, considering factors like the availability of each node to mitigate these potential issues. Additionally, in the proposed PBFT scheme, although each cluster has a CPN, the number of CPNs is a configurable value. Therefore, the security issue of CPNs can be mitigated by using multiple CPNs to structure the blockchain network [25]. Another method involves using agents to periodically monitor key nodes, such as the Cluster Primary Node [26]. These agent nodes are responsible for surveilling and managing key nodes participating in the blockchain network, enabling them to take immediate action if a problem is detected during monitoring. Although utilizing agent nodes may introduce some overhead, this does not affect the consensus process since these agents do not participate in the consensus process and primarily focus on monitoring and managing the key nodes. Additionally, in a private blockchain, agent nodes can be regarded as administrator nodes similar to clients, thus reducing the likelihood of malicious behavior.

The performance and effectiveness of the proposed PBFT scheme have only been verified in a simulation environment. This is an aspect that requires further improvement in our study. Consequently, it is necessary to analyze the performance and effectiveness of the proposed PBFT scheme in an actual blockchain network.

## 6. Conclusions and Future Works

In a blockchain network, consensus algorithms are crucial as they ensure all participants reach an agreement on consensus messages, including transactions, thus maintaining the integrity and security of the distributed ledger. PBFT, a consensus algorithm often used in private blockchains, provides reliability but encounters scalability challenges, as its performance can significantly decline with an increasing number of participating nodes.

This paper proposed an enhanced PBFT method through dynamic hierarchy management and location-based clustering to address the scalability issue. This method extends the typical PBFT and DLM-PBFT by organizing nodes into clusters based on geographical information, which accelerates the consensus process within clusters. Its primary advantage is the reduction in consensus processing time and communication overhead across the blockchain network. By optimizing communication paths and minimizing the travel distance of messages, the method significantly enhances scalability and efficiency, especially in networks with a large number of nodes.

Additionally, we have proposed a model for calculating the time required for each phase of the proposed PBFT protocol. Based on this model, we present a method to dynamically adjust the blockchain network’s hierarchical structure to minimize the estimated time for consensus. This takes into account variables such as the number of nodes in each group and the total number of nodes within each cluster. This dynamic management of the network’s hierarchy has been applied to the PBFT method we propose.

Simulations were conducted to compare the performance of the proposed PBFT method against that of the typical PBFT and DLM-PBFT. The proposed PBFT method demonstrated significant performance improvements based on the experimental results. It showed a processing performance enhancement of approximately 107% to 128% over the typical PBFT, and 11% to 99% over DLM-PBFT. The proposed PBFT method is expected to substantially reduce latency and enhance throughput in blockchain operations, offering a scalable solution for networks with a high volume of transactions. By optimizing node communication, it can maintain robust security while increasing overall network efficiency.

The performance and effectiveness of the proposed PBFT scheme have been verified through simulation. In future work, we plan to apply this scheme to an actual blockchain environment to further verify its performance and effectiveness. Additionally, we intend to research methods for minimizing the overhead associated with dynamic hierarchy management and location-based clustering.

## Figures and Tables

**Table 1:** Comparison of Consensus Algorithms.

Name	Communication Complexity	Clustering	Grouping	Layer Management
PBFT	$O(N^{2})$	No	No	No
G-PBFT	$O(N_{group}\times {N_{g_{node}}}^{2})$	No	Yes	No
SDMA-PBFT	$O(N\times K\times \log_{K}N)$	No	Yes	Yes
DLM-PBFT	$O(N\times K^{2})$	No	Yes	Yes
Proposed PBFT	$O(N_{cluster}\times N_{node}\times N_{g\_node}^{2})$	Yes	Yes	Yes

**Table 2:** Notations and their descriptions.

Notations	Descriptions
$N_{total}$	The total number of nodes in the blockchain network
$N_{node}$	The total number of nodes within one cluster
$N_{g\_node}$	The maximum number of nodes within a group
$N_{child}$	The maximum number of child groups for CPN and GPN
$N_{hierachy}$	The number of hierarchy layers within a cluster
$N_{cluster}$	The number of clusters in the blockchain network
BW	The average bandwidth of nodes participating in consensus
$L_{inter}$	The communication latency occurring when communicating with the outside of the cluster
$L_{intra}$	The communication latency between nodes within the cluster
$S_{block}$	Size of the consensus message
$S_{reply}$	Size of the agreement result message
$T_{verify}$	The time required to verify the message in the preparation phase
$T_{setBlock}$	Time required for Commit phase
TH	Maximum number of consensus iterations possible using the same hierarchy and group structures
$NC_{consensus}$	The current number of consensus executions
$T_{phase}$	Estimated time for each consensus phase [pre-request, request, preparation, etc.]
$T_{total}$	Total time required for consensus
$T_{total}\left(c,g,n\right)$	Total expected consensus time, where *c* represents the maximum number of child nodes that both CPN and GPN can have, *g* denotes the number of nodes composing the group, *n* denotes the total number of nodes within one cluster
$T_{expected}$	Expected consensus time
$GHI_{best}$	Information about the best grouping and hierarchy structures

**Table 3:** Experimental environments.

Name	Descriptions
OS	Ubuntu 20.04LTS
Mainboard	ASRock TRX40 CREATOR ASWin
CPU	AMD Ryzen Threadripper 3960X 24-Cores Processor (12 Cores, 24 Threads)
Memory	DDR4 64 GB (16 GB × 4)
Python	3.7.13
aiohttp	3.8.1

**Table 4:** Parameters used in the experiment and their settings.

Parameters	Descriptions
$N_{total}$	120, 160, 200, 240, 280, 300
$N_{g\_node}$	4
$N_{cluster}$	4
$N_{child}$	2, 4, 6, 8, 10, 12, 14, 16, 18, 20
$S_{block}$	1 MB
$L_{inter}$	0.063 s, 0.126 s, 0.252 s
$L_{intra}$	0.03 s

## Data Availability

Data are contained within the article.
